# Exploiting endovascular aortic repair as a minimally invasive method – Nine years of experience in a non-university hospital

**DOI:** 10.1016/j.ejro.2023.100522

**Published:** 2023-09-04

**Authors:** Lars Borgen, Kjartan Aasekjær, Øyvind Werpen Skoe

**Affiliations:** a Department of Radiology, Drammen Hospital, Vestre Viken Health Trust, Dronning gaten 28, 3004 Drammen, Norway; b Department of Surgery, Drammen Hospital, Vestre Viken Health Trust, Dronning gaten 28, 3004 Drammen, Norway

**Keywords:** Vascular, Aorta, Aneurysm, Interventional

## Abstract

**Background:**

At the introduction of endovascular aortic repair (EVAR) in 2013 in our non-university hospital, we established a quality registry to monitor our EVAR activity.

**Purpose:**

To observe if we over time were able to exploit EVAR as a minimally invasive method in an elective as well as emergency setting, and to monitor our treatment quality in terms of complications, secondary interventions and mortality.

**Material and methods:**

From November 2013 to March 2022, we treated 207 patients with EVAR, including six patients with rupture. Follow-up regimen was partly based on contrast-enhanced computer tomography, and partly on contrast-enhanced ultrasound in combination with plain radiography.

**Results:**

During the observation period, the method of anesthesia changed from general, via spinal, to local anesthesia. The groin access changed from surgical cut down to percutaneous and the median length of postoperative stay decreased from 3 days to 1 day. EVAR on ruptured aneurysm was done for the first time in 2019. Endoleak was detected in 85 patients (42%) and 37 patients (18%) had one or more secondary interventions, of which 85% were endovascular. Estimated five-year survival was 72% in patients below 80 years of age and 45% in patients 80 years or older.

**Conclusion:**

Nine years of experience enabled us to exploit EVAR’s advantages as a minimally invasive method in an elective as well as emergency setting. Complications, secondary interventions and survival rates in our low volume non-university hospital matches results from larger vascular centers.

## Highlights

• A long learning curve: it may take years to exploit the advantages of EVAR.
• EVAR as a minimally invasive procedure – advantages take years to exploit.
• EVAR can be performed safely in small vascular centers.
• Smaller non-academic vascular center show comprehensive EVAR treatment results.

## 1 Introduction

As a minimally invasive method for the treatment of aortic aneurysms, endovascular aortic repair (EVAR) has been a game changer. Compared to open surgery, EVAR has potential benefits for the patient related to limited procedural trauma, less postoperative discomfort, faster mobilization and reduced length of stay [1]. Several studies have also shown better perioperative survival for ruptured abdominal aortic aneurysm (AAA) with EVAR compared to open repair (OR) [2]. On the other hand, long term outcome for EVAR has shown inferior results compared to OR [3], and EVAR requires lifelong patient follow-up.

Exploiting all the potential benefits of EVAR may not be possible from the introduction of the method in a vascular center. Experience over time, acquiring technical skills, establishing adequate protocols, necessary equipment and interdisciplinary relations are all factors influencing the extent to which EVARs potential benefits are realized [4], [5]. Performing EVARs in an emergency setting is a complex and demanding task requiring experience from the elective setting and training over time [6].

We introduced EVAR in 2013 at Drammen hospital, a non-university hospital in Norway with a catchment population of about 480.000 people. At the time of introduction, we established a local quality registry to monitor our development and quality of practice, which included patient demographics, procedural data, endoleak detection, secondary procedures and mortality.

Interventional radiologists and vascular surgeons participate in the EVAR treatment on equal terms in our hospital, and inclusive interdisciplinary teamwork has been a priority. Adequate C-arm angiography equipment, Ziehm Vision RDF (Ziehm Imaging GmbH, Nurenberg, Germany) placed in the operating theater of the vascular surgery unit was purchased in 2018. Before this, all EVAR procedures were performed in the angiography laboratory located in the X-ray department.

In this observational study, we wanted to investigate how our 9 years of experience with EVAR in a non-university hospital enabled us to take advantage of EVAR as a minimally invasive procedure, and to compare our rates of complications, secondary interventions and survival to those found in the literature.

## 2 Material and methods

### 2.1 Study design

We performed a retrospective study of our patients treated with EVAR during the period from November 2013 to March 2022. This study was approved by the data protection officer in our institution. Informed consents were obtained from all participants in writing.

### 2.2 Patient selection and follow up

Aneurysm treatment was indicated if the aneurysm diameter reached 55 mm for males or 50 mm for females, if their aneurysm was symptomatic, or if the diameter increased by more than 5 mm over the preceding 6 months. Patients of age ≥ 70 years or deemed unfit for OR were offered EVAR. For asymptomatic AAA at our hospital, the proportion of patients treated with EVAR vs. OR has been unchanged about 40% vs. 60%.

### 2.3 Statistical analysis

Statistical analysis was conducted using IBM SPSS statistic software (SPSS Inc., Chicago, IL, USA). Median (range) was used for non-parametric distributions. Kaplan-Meyer survival curves were utilized to estimate freedom from secondary procedures as well as overall survival.

## 3 Results

### 3.1 Change in follow up regimen

Initial follow-up regimen consisted of multiphased contrast-enhanced computer tomography (CECT) at one and 12 months, and thereafter yearly CECT. However, during the second half of the observation period, CECT has partially been replaced by contrast-enhanced ultrasound (CEUS) in combination with abdominal radiography at 24 months and later (Table 1).

**Table 1:** Percentage of patients at EVAR controls examined by CECT or CEUS in combination plain radiography.

EVAR control after implantation										
Months	1	12	24	36	48	60	72	84	96	108
CECT (%)	100	78	46	24	25	14	14	0	50	0
CEUS (%)	0	26	54	66	75	86	86	100	50	100

### 3.2 Patient demographics

Table 2 shows patient demographics. Of 201 intact aneurysms, 185 were true abdominal, nine were true iliac aneurysms and seven were pseudoaneurysms related to an older surgical anastomosis. Six patients presented with a rupture: four abdominal aneurysms of which two had an aortoenteric fistula, and two iliac aneurysms.

**Table 2:** Patient demographics.

Patient demographics	n	%
Total	207	
Gender (M:F)	175:32	(85:15)
Age (range)	76 years (52–92 years)	
Cardiac disease	116	56
Hypertension	106	51
Malignancy	67	32
Chronic pulmonary disease	54	26
Diabetes mellitus	32	16
Cerebrovascular disease	31	15
Renal failure stadium III	56	27
Renal failure stadium IV	7	3
Active or former cigarette smoking	161	78
Former abdominal surgery	69	33

### 3.3 Configuration and type of stentgrafts

We treated 187 patients (90%) with bifurcated aortobiiliac stentgraft, nine patients (4%) with aortic tube and seven patients (3%) with aortouniiliac stentgraft supplemented with femorofemoral bypass. Two patients (1%) received an iliac branched stentgraft, and two patients (1%) had a chimney EVAR. The Endurant stentgraft system (Medtronic, Santa Rosa, CA, USA) stentgraft has been our main stentgraft and was used in 141 patients. Partially due to vascular anatomy and partially to tender issues, Cook Zenith Alpha (Limerick, Ireland), Gore Excluder (W.L. Gore & Associates, Newark, DE, USA), Incraft (Cordis, Zurich, Switzerland) and Ovation (Endologix, Hertogenbosch, Netherlands) have also been used.

### 3.4 Analgesia, groin access and length of postoperative stay

**Fig. 1:**
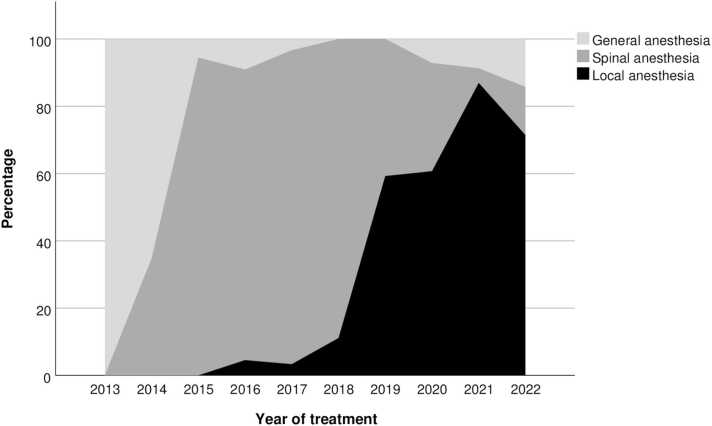
Method of analgesia during the observation period.

During the first year, the standard method of analgesia was general anesthesia for all patients, with total control of the patient and respiratory movements. However, we changed to primarily spinal anesthesia during the middle of the period, and finally adopted local groin anesthesia as our method of choice for pain prevention during the last four years (Fig. 1).

**Fig. 2:**
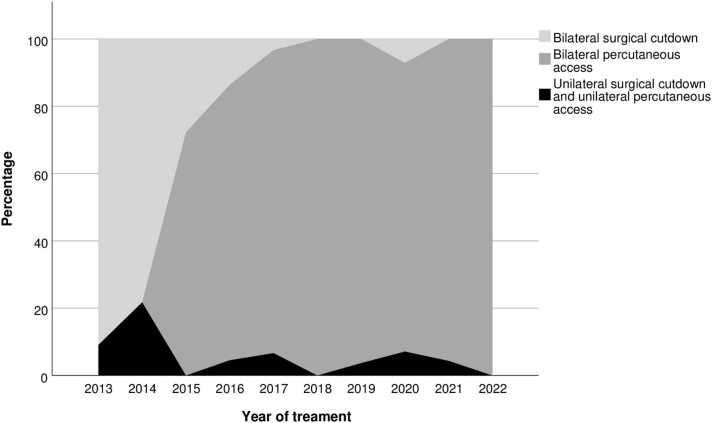
Vascular access during the observation period.

Groin access was initially achieved by surgical cutdown of the femoral arteries. During the observation period, we transitioned to percutaneous access as our primary approach, using the closure device Proglide (Abbott Vascular, USA) (Fig. 2).

**Fig. 3:**
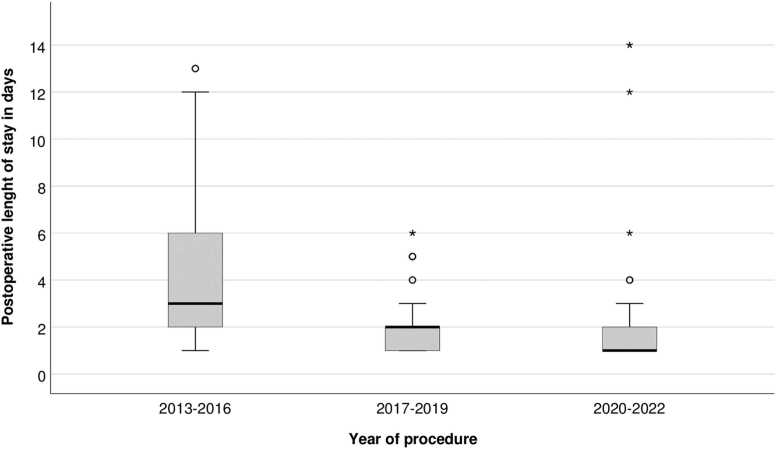
Postoperative length of stay for elective EVAR.

Median length of postoperative stay for elective EVAR patients was reduced from 3 days to 1 day during the period (Fig. 3), while EVAR patients with a ruptured aneurysm had a median postoperative length of stay of 13 days (1–16 days). The length of postoperative stay for AAA patients treated with OR at our hospital remained unchanged, with a median of 8,5 days (2–72 days) for elective patients and 9 days (0–33 days) for ruptures.

### 3.5 EVAR on ruptured aneurysms

Until 2018, we performed EVAR exclusively on intact aneurysms, but during the last four years of the observation period, six patients underwent EVAR for ruptured aneurisms.

### 3.6 Complications

Among 91 groins with surgical cutdown, two (2%) needed immediate reoperation due to hematoma and no femoral pseudoaneuryms were seen. Among 323 percutaneous groins, five (2%) needed immediate cutdown due to lack of hemostasis. Four (1%) femoral pseudoaneurysms were seen, of which two required surgery and two were observed. We did not observe any common femoral artery stenosis.

Endoleak was detected during follow up in 85 patients (41%). In 47 patients (23%), the endoleak ceased spontaneously, while in 24 patients (12%), the endoleak appeared later than on CECT at 1 month. Type I endoleak was seen in 10 patients (5%), type II endoleak was seen in 74 patients (36%), and type III endoleak was seen in one patient (0,5%).

Two patients (1%) had acute lower limb ischemia following the EVAR procedure, while 12 patients (6%) developed lower extremity ischemia due to limb kinking or thrombus formation during follow up; among these seven patients (3%) had an occluded stentgraft limb.

Five patients (2%) were diagnosed with a graft infection. Among these, two patients had an aortoenteric fistula as the cause of infection. One patient had a total colectomy two weeks prior to EVAR, and was treated for an impending rupture. Two patients had a stentgraft infection at 2 months and 4 years respectively, with no obvious cause.

### 3.7 Secondary procedures

**Fig. 4:**
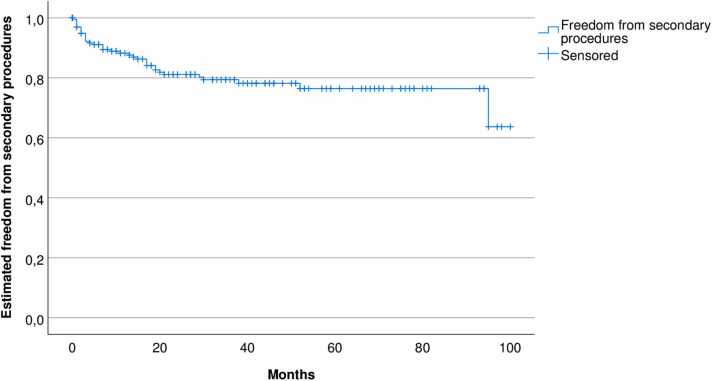
Estimated freedom from secondary interventions, Kaplan-Meyer diagram.

One or more secondary procedures were done in 37 patients (18%). Estimated freedom from secondary procedures is shown in Fig. 4. Of totally 80 secondary procedures, 68 were endovascular and 12 surgical. The indications for performing the secondary procedures was endoleak for 22 patients (11%), ischemia for 12 patients (6%), pseudoaneurysm for two patients (1%) and infection for one patient (0,5%). Patients with endoleak type II and aneurysm growth > 5 mm were treated.

EVAR explantation was performed in three patients. One patient had a secondary rupture due to endoleak type II, while scheduled for his second embolization attempt. One patient had a refractory type II endoleak with sac enlargement, and one patient had an axillobifemoral bypass due to graft infection.

### 3.8 Mortality

**Fig. 5:**
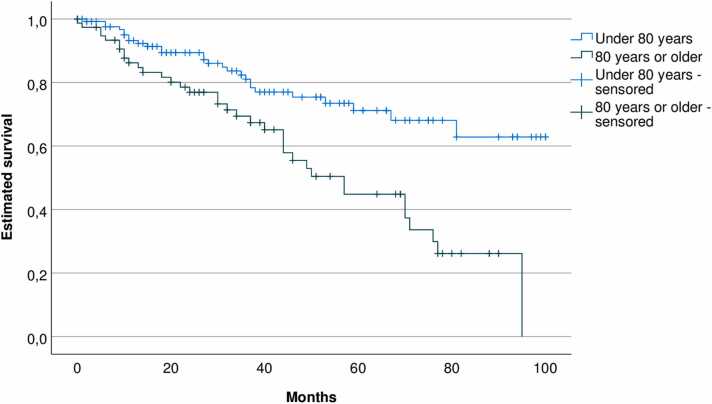
Estimated survival, Kaplan-Meyer diagram.

Thirty-day mortality for intact and ruptured aneurysms was 0% and 30% respectively. Estimated long-term survival for 201 patients with intact aneurysm is shown in Fig. 5. For patients < 80 years, two- and five-year estimated survival rate were 90% and 72% respectively. For octogenarians or older, the corresponding estimated survival rates were 78% and 45%.

## 4 Discussion

In this study, we have shown that years of experience enabled us to go from open to percutaneous access, from general via spinal to local anesthesia, and to shorten the length of postoperative stay after EVAR. Endoleaks were frequent and 18% of the patients needed one or more secondary interventions. For patients treated with elective EVAR, 30-day survival was excellent while long-term survival was moderate.

### 4.1 Change in follow up regimen

For patients with stable aneurysms and no known endoleak, or renal failure stadium III-IV, controls were mostly done by CEUS. For obese patients unfit for ultrasound, CECT remained the preferred modality regardless of aneurysm sac development and endoleaks. We have increased control intervals up to 24 months for patients with stable or shrinking aneurysms.

### 4.2 Analgesia, groin access and length of postoperative stay

Through the transformation from general, via spinal, to local anesthesia we made use of one of EVAR’s advantages as a minimally invasive method. Cardiovascular comorbidity is common in these patients, making them vulnerable to the circulatory side effects of general and spinal anesthesia - this being especially relevant for already hypotensive patients with a rupture. Furthermore, antithrombotic treatment may contraindicate spinal anesthesia. In our experience, local anesthetics with awake patient works well, with adequate patient cooperation and pain relief. This harmonizes with the guidelines of the European society for vascular surgery (ESVS) [7], where EVAR in local anesthetics is recommended.

During the initial phase of performing EVAR, we found that access by surgical cut down allowed for optimal control of groin closing, presumably minimized the risk of hematomas. However, after gaining more confidence with EVAR, we found it appropriate to advance to percutaneous access. Gimzewska et al. reported similar complication rates for cutdown and percutaneous access [8], and this correlates with our results, demonstrating equal rates of hematomas for cutdown and percutaneous access.

During the observation time, we were able to reduce the postoperative length of stay after EVAR. Thus, comparing with OR, the length of stay was 7,5 days shorter in recent years for EVAR patients. Building confidence in the percutaneous groin closure technique and establishing post procedure routines have enabled us to exploit the EVAR advantage of short hospital stay. Reduced intensive care unit and hospital length of stay are well known advantages of EVAR compared to open surgery [9], [10]. EVAR in an outpatient setting can be done safely [11], but we find it appropriate with 1 day of observation after the procedure, partially due to the patients preferring not to be discharged on the day of the procedure.

### 4.3 Aneurysm size on follow up

Of 151 patients with one or more follow up aneurysm measurements, 75% had aneurysm sac shrinkage. 22% had an increase of aneurysm diameter at some point during observation, while 5% remained unchanged.

### 4.4 EVAR on ruptured aneurysms

EVAR on ruptured aneurysms was first done at our hospital in 2019. Adequate C-arm angiography equipment in place in the vascular surgery operating theater from late 2018 lowered the threshold for emergency EVAR, since the conditions for possible conversion to open surgery then were considered adequate. Besides equipment issues, establishing adequate protocols and competence in all involved personnel required years of experience in a center with limited EVAR volume. This learning curve is illustrated by the fact that in the year following the observation time for our study, from April 2022 to March 2023, we treated three ruptured AAA with EVAR and five with OR. Hence, our present proportion of EVAR vs. OR for ruptured AAA is now 38% vs 62%.

### 4.5 Complications

The rate of endoleaks, with type II dominating, is as expected in our study population. The rate of type II endoleaks varies in the literature from 5% to 62%, but most studies report a rate of 10–20% [12]. The reported number of endoleaks will necessarily depend on the observation time [13] and the sensitivity of the follow up regimen [14].

Our rates of lower limb ischemia and limb occlusions are in concordance with other studies. Maldonado et al. reported such ischemic complications in 9% of 311 patients after EVAR, limb occlusion being the dominating cause of ischemia [15]. The graft infection rate in our material was 2,5%, while the rate of infection after EVAR ranges from 0.3% to 1% in the literature [16], [17]. Our slightly higher infection rate is due to the two aortoenteric fistulas and the one patient recently having undergone colectomy. These are rare cases that we may not encounter again for quite some time.

### 4.6 Secondary procedures

The incidence of secondary procedures for endoleaks, ischemia and access complications in our material is in concordance with the literature [18], [19]. Of all our secondary procedures, 85% were endovascular. Hence, the majority of patients continued to have the benefit of EVAR as a minimally invasive procedure, as there was no need for open surgery. In our material, 2.5% of EVAR patients were converted to OR, harmonizing with low conversion rates after EVAR in other studies. Moulakakis et al. reported a conversion rate of 1,9% in a review of 12.236 patients [20].

### 4.7 Mortality

In our population, the 30-day mortality after elective EVAR was 0%, which is in concordance contemporary studies showing a 30-day mortality rate of around 1% [21]. Estimated five-year survival in our material concurs with mortality rates reported by others [22]. Primarily, the long-term survival is dictated by comorbidity rather than aneurysm related pathology. On the other hand, a low 30-day mortality in the elective setting in our material may reflect adequate technical and procedural quality standards.

### 4.8 Strengths and limitations

This is a retrospective study with no control group for comparison. Hence, direct comparison against open surgery cannot be conducted. Furthermore, we have not separated patients treated within and without instructions for use, which may influence on treatment results. On the other side, our material represents real world data from small volume non-university hospital, complementing other studies, often from larger academic vascular centers.

## 5 Conclusion

In conclusion, several years of experience with EVAR enabled us to exploit the advantages of EVAR as a minimally invasive procedure in the elective as well as in the emergency setting. Complications, secondary interventions and survival rates in our non-university hospital are in concordance with the literature.

## Funding

This research did not receive any specific grant from funding agencies in the public, commercial, or not-for-profit sectors. This study had no external funding; all authors did this research as part of their job at Drammen hospital.

## Ethical statement

The present work has been carried out in accordance with The Code of Ethics of the World Medical Association (Declaration of Helsinki) and is in line with the Recommendations for the Conduct, Reporting, Editing and Publication of Scholarly Work in Medical Journals. Moreover, the work aims for the inclusion of representative human populations (sex, age, and ethnicity) as per those recommendations. The terms sex and gender are used correctly. This study was approved by the data protection officer in our institution. Informed consents were obtained from all participants in writing. The privacy rights of human subjects have been always observed.

## CRediT authorship contribution statement

**Lars Borgen:** Conceptualization, Methodology, Investigation, Data curation, Writing – original draft, Project administration. **Kjartan Aasekjær:** Conceptualization, Investigation, Writing – review & editing. **Øyvind Werpen Skoe:** Conceptualization, Investigation, Writing – review & editing.

## Declaration of Competing Interest

The authors declare that they have no known competing financial interests or personal relationships that could have appeared to influence the work reported in this paper.
